# The Immunohistochemistry Profile of Lymphocytic Gastritis in Celiac Disease and Helicobacter Pylori Infection: Interplay between Infection and Inflammation

**DOI:** 10.1155/2007/81838

**Published:** 2007-11-20

**Authors:** Efrat Broide, Judith Sandbank, Eitan Scapa, Nimrod Alain Kimchi, Michael Shapiro, Aaron Lerner

**Affiliations:** ^1^Institute of Gastroenterology, Assaf Harofeh Medical Center, Sackler School of Medicine, University of Tel-Aviv, Zerifin 70300, Israel; ^2^Institute of Pathology, Assaf Harofeh Medical Center, Sackler School of Medicine, University of Tel-Aviv, Zerifin 70300, Israel; ^3^Pediatric Gastroenetrology and Nutrition Unit, Carmel Medical Center, Rappaport School of Medicine, Technion-Israel Institute of Technology, Haifa 32000, Israel

## Abstract

Lymphocytic gastritis (LG) is associated with helicobacter pylori (Hp) and celiac disease (CD). We aimed to clarify the relationship between Hp infection and CD by defining a unique histopathology profile of LG in these two diseases. Forty patients who underwent upper endoscopy were divided into four groups: eight controls, ten active CD patients without Hp, twelve CD negative with Hp, and ten active CD with Hp infection. Antral samples were assessed by immunohistochemical staining for CD20, CD3, CD4, CD8, CD57, CNA42, and Ki67 for lymphoid aggregates, intraepithelial lymphocytes (IELs) number, density of lamina propria (LP) lymphocytes, and inflammatory glandular involvement. Only IELs positive for CD3 and CD8 were increased significantly in CD patients with or without Hp infection. Hp did not contribute to the number of CD8 IELs. In complicated cases with Hp and suspicious for CD, the number of CD8+ IELs hints toward a CD rather than Hp infection.

## 1. INTRODUCTION

Helicobacter pylori (Hp) infection may cause several clinical manifestations, ranging from asymptomatic to significant gastroduodenal disease including ulcer, bleeding, perforation, and adenocarcinoma [1]. No pathogenic mechanism is known to explain the above diversity.

Celiac disease (CD) is a T-cell-mediated disorder of the small bowel triggered by gluten in susceptible subjects. The damage is not confined only to the small bowel but may affect the gastric mucosal structure and function [2]. In both conditions, Hp and CD, a systemic humoral immune response is detected, but much interest has been focused on the local immune inflammatory reaction.

Lymphocytic gastritis (LG) was initially characterized by Haot et al. in 1986 [3, 4]. It is defined by the presence of >25 intraepithelial lymphocytes (IELs) (surface and upper foveolar epithelium) per 100 epithelial cells, without taking into account the mononuclear inflammatory cell infiltration of the LP. This condition may be recognized endoscopically as varioliform gastritis, nodularity, hypertrophic gastropathy, and aphtous erosions [5, 6]. A similar histological entity has been reported in association with a variety of gastric infections, inflammatory diseases, and in autoimmune disorders affecting the gastric mucosa including CD, in adults [7–9], as well as in pediatric patients [10–12]. Additionally, Hp is a major etiology for LG, extensively documented in adults [13, 14] and children [15, 16]. Furthermore, investigations of the relationship between Hp infection and CD have yielded conflicting results [9, 17], probably because of the different prevalence of Hp in the populations studied. Others have focused on the Hp-related LG in CD [6, 8] and recently on the link between anemia, Hp, and CD [18]. The pathogenetic relationship between CD, Hp and LG is even more complicated since gastric Hp existence attenuates duodenal lesions in CD patients [19].

It is generally accepted that the major cytokine response to Hp and CD has a T-helper 1 (Th1)-type profile [20]. The exact nature of the cellular response contributing to this inflammatory profile has not been determined, and less so in children. Conflicting results exist in the literature on the lymphocytic subpopulations, quantity and quality, infiltrating the gastric mucosa in CD and Hp.

As LG is a morphologic endpoint of numerous etiologies, the literature is ripe with controversy [9] and since extensive characterization of the lymphocytic populations infiltrating the gastric mucosa in CD and Hp infected children is lacking, the present study was undertaken. The aims were to look for additional quantitative and qualitative histological features, to define the immunophenotype of the gastric mucosa of the two most prevalent etiologies of pediatric LG (CD and Hp infection). Our secondary aim was to demonstrate a unique histopathology profile of LG by immunohistocemistry staining, trying to clarify the interrelationship between Hp and CD in the pediatric population, thus shedding new light on the two entity's enigma.

## 2. PATIENTS AND METHODS

### 2.1. Patients

Forty patients referred for endoscopy due to upper gastrointestinal symptoms (mostly recurrent abdominal pain, or iron deficiency anemia) were included in the study. Patients were divided into 4 groups group1 is eight normal controls (without CD nor Hp infection); group 2 is ten patients with new active CD without Hp infection; group 3 is twelve Celiac negative patients infected by Hp; and group 4 is ten new active CD patients infected by Hp (Table 1). None of these patients had any other gastrointestinal underlying disease including evidence for food allergy, giardiasis, or inflammatory bowel disease. The diagnosis of CD was based on the accepted histological findings [9] supported by positive serology for CD (antiendomysial and antitissue transglutaminase antibodies) [10]. All CD patients were under normal gluten containing diet at the time of diagnosis. Hp status was assessed according to conventional biopsy-based criteria plus positive urease test. All patients underwent esophago-gastro-duodenoscopy using GIF-xp 20 endoscope; Pentax, Tokyo, Japan. At least 6 biopsies were obtained: 3 from the second part of the duodenum for diagnosing or exclusion of CD, and 3 from the antrum; one for quick urease test and two for histologic examination. The local ethical committee approved the study.

### 2.2. Histopathological studies

Sections obtained from gastric biopsies were immediately fixed in buffered formalin and embedded on edge in paraffin. Serial 3 μm thick sections were obtained for histological and immunohistochemical examinations. Sections were stained with hematoxylin-eosin and with Giemsa. The diagnosis of LG was established if 25 lymphocytes per 100 gastric epithelial cells infiltrated the surface epithelium. Furthermore, we estimated the extent to which the inflammatory infiltrate involved the LP and the mucosal glands. In addition, the number of mucosal and submucosal lymphoid aggregates were counted. To clarify the nature of these inflammatory cells, additional paraffin sections were immunostained with several antibodies.

### 2.3. Immunohistochemical studies

Immunohistochemistry was performed using standard methods. Three μm sections were prepared from formalin-fixed, paraffin-embedded tissue blocks, air dried, and subjected to deparaffinization with xylene and absolute alcohol. The immunoperoxidase stains were performed using a panel of antibodies that included CD20, CD3, CD4, CD8, CD57, CNA42, and Ki67 (Table 2), with appropriate dilutions as recommended by the manufactures, using the Ventana ES autoimmunostainer and the iVIEWDAB detection kit from Ventana. The sections were counterstained with Mayer's hematoxylin.

### 2.4. Inflammation grading

The gastric IELs were counted on the hematoxylin-eosin stains per 100 consecutive gastric surface mucosal cells with a X400 magnification (objectiveX10).

The density of subepithelial lymphocytes was determined semiquantitatively using a 4 tier grading system, according to the percentage of the area in the LP infiltrated by the inflammatory cells. Grade 0 is if less than 5% of the area was infiltrated by inflammatory cells, grade 1 is between 5–30%, grade 2 is between 30–60%, and grade 3 is if *>*60% of the area was infiltrated. In order to evaluate the inflammatory glandular involvement, glands containing inflammatory cells were counted out of a fixed total number of superficial glands; calculated as the percent of involved glands.

### 2.5. Statistical analysis

The data were analyzed using BMDP [11]. Continuous variables were compared across groups using analysis of variance (ANOVA) with Bonferroni's correction for multiple comparisons. Discrete (semi quantitative) variables were compared using Kruskal-Wallis nonparametric one-way analysis of variance, with multiple comparisons. A P-value less or equal than 0.05 was considered significant.

## 3. RESULTS

Demographic data of the four examined groups of patients are summarized in Table 1. The mean age of all groups and gender's ratio were not statistically significant different. In CD as well as in Hp gastritis patients, increased numbers of IEL compatible with the diagnosis of LG (45.5±18.5 per 100 gastric epithelial cells) compared to patients without LG (10.8±4.5 per 100 gastric epithelial cells), were observed.

The highest proportion of LG (50%) was found in CD patients without Hp infection, followed by patients with CD infected by Hp (20%), Hp positive patients without CD (8.3%) and none in the control group.

### 3.1. Lymphoid aggregates

The distribution and total number of lymphoid aggregates in the antrum among the four different groups are shown in Table 3. Patients infected with Hp had the highest number of lymphoid aggregates irrespective of the CD status. Three CD patients with Hp and nine Hp infected patients without CD had more than 2 lymphoid aggregates.

### 3.2. Immunohistochemical results

**Figure 1:**
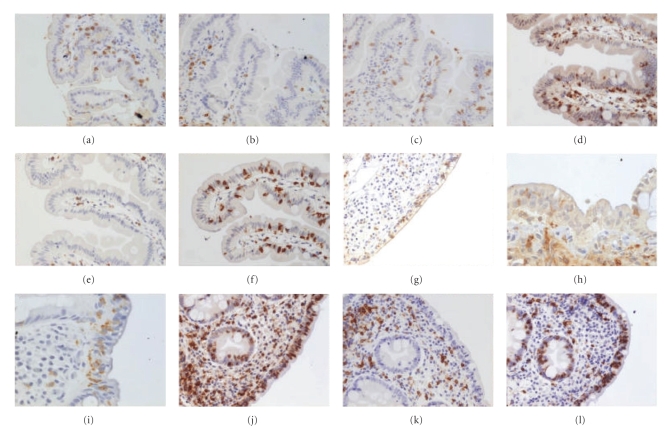
Immunohistochemical staining for CD3, CD4, and CD8 in the four different groups. Controls: (a)-CD3, (b)-CD4, (c)-CD8 (all o.mX200); Hp+/CD-: (d)-CD3 (o.mX400), (e)-CD4 (o.mX200), (f)-CD8 (o.mX200); Hp-/CD+: (g)-CD3 (o.mX200), (h)-CD4 (o.mX400), (i)-CD8 (o.mX400); Hp+/CD+: (j)-CD3, (k)-CD4, (l)-CD8 (all o.mX200).

The mean number of Intraepithelial lymphocytes (IELs) positive for CD3 was increased significantly in CD patients with or without Hp infection compared to controls [p≤0.01,p≤0.05 respectively], (Table 4). Similarly the mean number of CD8+ IELs was increased significantly in CD patients with or without Hp infection compared to controls (p<0.05) (Figure 1). Hp infection did not contribute to the number of CD8+ IELs. In the Hp infected group without CD, the number of CD8+ IELs was not significantly different from the normal controls [Table 4]. A slight increase in CD8+ lymphocytes was noted in the LP and intra mucosal glands in the CD group patients compared with all the other groups, although these results did not reach statistical significance.

CD4+ lymphocytes were slightly increased in the LP and in the mucosal glands of the Hp+ patients compared with controls and CD cases.

No statistical significant differences were found for the number and distribution of B lymphocytes (CD20+), Natural killer (NK) lymphocytes (CD57) and follicular dendritic cells (CNA42) within all different examined compartments (IELs, LP and glands). The proliferation marker Ki67 did not contribute to the results.

## 4. DISCUSSION

In order to investigate the differential contribution of Hp infection to LG in an inflammatory condition like CD, the gastric tissue inflammatory subpopulation profile was investigated, by immunohistochemistry, and compared between the 4 groups of patients. Past studies focused mainly on the surface epithelial infiltrate as well as on the superficial pit gastric epithelium invasion by lymphocytes, and to a lesser extent on the infiltrate in the LP and in the gastric glands.

The main finding of the present study was the highest prevalence LG in pediatric CD^+^Hp^-^ followed by CD^-^Hp^+^.

Forty five percent of CD patients are estimated to have LG like in our study [8, 12]. However, LG appears to be similarly frequent in Hp positive children with and without CD [13]. Previous studies demonstrated that LG was found to be more common in Hp positive children without CD than in Hp negative children without CD. The IELs were almost exclusively T cells [14]. There are still controversial results on the contribution of CD and Hp infection to LG. Moreover, the association between these pathologies to LG is not well established [13–15]. Lymphocytic gastritis was reported in 36–45% of children with CD [6, 10, 13], and disappears after a gluten free diet. Hp infection is less frequently found in patients with LG [13%] than with the usual chronic antral gastritis [65–90%] [13]. CD is considered to be a population at greatest risk for LG compared to Hp affected patients.

Our study confirmed the published results of an increased number of IELs in both CD and Hp infected patients. The most prominent LG was found in CD patients without Hp infection. Surprisingly, CD patients with associated Hp infection showed a lower rate of LG. This evidence might be explained by the well known limited roll of bacterial infection in cytotoxicity. Hp convergent the immune response towards Th2 response and suppress the Th1 immune response.

Previous investigators have published several sets of data concerning the composition of the lymphocytic infiltrates in the different diseases [16–20]. Drut et al found that LG in pediatric CD patients contains a peculiar CD3, CD7 and CD8 intraepithelial lymphocyte population, that is not associated with the presence of CD4, CD20, CD56 and CD57 IELs [21]. In Hp gastritis there is an increased number of mononuclear cells in the gastric LP, including B and T lymphocytes, plasma cells, macrophages and mast cells. Lymphoid aggregates are particularly characteristic of Hp infection [22]. It has been shown that Hp stimulates B lymphocytes and causes an increase in their numbers predominantly in the LP [23]. Although immunity against Hp infection appears not to be dependent on B cells, the role of T cells still remains to be clarified [24]. It appears however that the B cell proliferation might be driven by activated lymphocytes (CD4+ cells) that might recall and activate mononuclear phagocytes.

Bedoya et al. demonstrated that the cellular response includes an innate nonspecific response represented mainly by polymorphonuclear cells and macrophages, as well as a T cell response with abundant positive staining with anti-CD8 antibodies, was observed indicative of a predominance of suppressor/cytotoxic T lymphocytes both in the LP and in the epithelium [22].

Similarly we also showed the presence of CD3+ IELs in both diseases (CD; Hp infection). Despite a potential additive effect between these two pathological processes, it is impossible to differentiate between them, based only on the number of the CD3+ IELs in the antral mucosa. CD3 staining is a pan T lymphocyte marker and does not differentiate between CD4 helper and CD8 suppressor/cytotoxic cells, therefore we aimed to characterize the T cell subsets immunophenotype (CD4+, CD8+, CD57).

In our study the CD8+ IELs were significantly higher in CD patients (20/100 epithelial cells), compared to controls (1/100 epithelial cells) or Hp infected patients without CD (2/100 epithelial cells). We assume that in undiagnosed patients with histological features compatible with LG, higher counts of CD8+ IELs may imply that the diagnosis is CD rather than Hp gastritis. Our findings support the published data that CD8+/CD4− IELs are involved significantly in the pathogenesis of CD [25].

In our study, the low expression of CD57 in the lymphoid cells, both in the epithelium, in the LP as well as in the mucosal glands indicate that NK cells may play a negligible role in these two pathologies.

We also tried to differentiate between these two causative agents of LG by looking at the proliferation marker, Ki67. Although both conditions were associated with a prominent adaptive immune activity, no increase in the proliferation index of the surface epithelium was demonstrated in these pathologies.

Dendritic cells (CNA42+) which serve as professional antigen presenting cells did not show an increased expression in both pathologies. This may indicate that the process of antigen presentation occurred in the lymph nodes. This notion is supported by the absence of CD4 (T helper) cells in the immune cell infiltrates found in the affected tissues. Thus, in the infected area we observed mainly the effector CD8 lymphocytes.

In addition to all the above, we confirmed the published results that the highest number of lymphoid follicles was observed in a similar proportion of children with or without CD, who were Hp positive independent of the presence of LG.

The inflammatory infiltrate in the LP and in the mucosal glands does not contribute to the differentiation between these diseases.

In summary, our study aimed to explore the contribution of CD and Hp infection to LG and to characterize the different immunoprofiles of the gastric inflammatory cells involved in these diseases. We were looking for an applicable histological tool that might differentiate between cases of CD and Hp infection with overlapping clinical and histological features.

We suggest that in very young patients infected by Hp and suspicion for having atypical CD (negative serology with increased IELs with normal villous architecture-Marsh I classification) with a debatable diagnosis of CD versus Hp infection, the number of CD8+ IELs in the antrum might hint toward the diagnosis of CD rather than Hp infection, and the number of lymphoid follicles directs toward the diagnosis of Hp infection. Thus, it is important to include immunohistochemical analysis of CD8 lymphocytes in the antrum in undefined cases of CD.

## Figures and Tables

**Table 1:** Demographic data.

Group	Mean age ± SD yrs (range)	Male/female	Total no.
Controls	12.5±5.63 [3–18]	2/6	8
CD+/Hp-	9.85±10.78 [1.5–38]	1/9	10
CD-/Hp+	15.66±5.08 [8–28]	3/9	12
CD+/Hp+	10.9±3.93 [5–17]	2/8	10
P value	NS	NS

**Table 2:** Antibodies used for immunohistochemical evaluation.

Ab	Source	Clone no	Ag retrieval	Dilution
CD3	DAKO	Polyclonal	Citrate buffer	1:50
CD20	DAKO	L26	Citrate buffer	1:400
CD4	Zymed	ZT-17	Citrate buffer	None
CD8	DAKO	C8/144B	Citrate buffer	1:40
CD57	Novocastra	NK-1	Citrate buffer	1:50
Ki67	DAKO	MIB-1	Citrate buffer	1:200
CNA42	DAKO	CNA42	Citrate buffer	1:75

**Table 3:** Lymphoid aggregates in antrum.

					Significance between groups					
	Group 1 controls	Group 2 CD+/Hp-	Group 3 CD-/Hp+	Group 4 CD+/Hp+	[1-2]	P[1–3]	P[1–4]	P[2-3]	P[2–4]	P[3-4]
Mean no.	0.125	0.900	3.75	2
SD	0.354	0.738	2.417	1.764	NS	<0.01	NS	<0.05	NS	NS
Minimum	0	0	0	0
Maximum	1	2	7	5

**Table 4:** Mean intraepithelial lymphocytes number (immunohistochemistry staining).

					Significance between groups					
Imunohistochemical staining	Group 1 controls	Group 2 CD+/Hp-	Group 3 CD-/Hp+	Group 4 CD+/Hp+	[1-2]	P[1–3]	P[1–4]	P[2-3]	P[2–4]	P[3-4]
CD3 [mean ± SD]	1	16±7.2	3.2±2.2	14±4.1	<0.05	NS	<0.01	NS	NS	<0.05
CD8	0	20.2±10.1	2.2±2.1	16.8±8.8	<0.05	NS	<0.05	<0.05	NS	<0.05
Ki67	0	2.8±2.3	0	2±1.6	NS	NS	NS	NS	NS	NS

## References

[1] Farrell RJ, Kelly CP (2006). Celiac sprue and refractory sprue. *Sleisenger & Fordtran's Gastrointestinal and Liver Disease*.

[2] Graham DY, Sung JY (2006). Helicobacter pylori. *Sleisenger & Fordtran's Gastrointestinal and Liver Disease*.

[3] Haot J, Jouret-Mourin A, Wallez Z (1986). Anatomoclinical study of a series of chronic gastritis characterized by intraepithelial lymphocytic infiltration. *Acta Endoscopica*.

[4] Haot J, Delos M, Wallez L, Hardy N, Lenzen B, Jouret-Mourin A (1986). Intraepithelial lymphocytes in inflammatory gastric pathology. *Acta Endoscopica*.

[5] Rubin CE, Brandborg LL, Phelps PC, Taylor H (1960). Studies of celiac disease, 1.The apparent identical and
specific nature of the duodenal and proximal jejunal lesion in celiac disease and idiopathic sprue. *Gastroenterology*.

[6] Wolber R, Owen D, DelBuono L, Appelman H, Freeman H (1990). Lymphocytic gastritis in patients with celiac sprue or spruelike intestinal disease. *Gastroenterology*.

[7] Glickman JN, Antonioli DA (2001). Gastritis. *Gastrointestinal Endoscopy Clinics of North America*.

[8] Diamanti A, Maino C, Niveloni S (1999). Characterization of gastric mucosal lesions in patients with celiac disease: a prospective controlled study. *American Journal of Gastroenterology*.

[9] Dewar DH, Ciclitira PJ (2005). Clinical features and diagnosis of celiac disease. *Gastroenterology*.

[10] Hill ID (2005). What are the sensitivity and specificity of serologic tests for celiac disease? Do
sensitivity and specificity vary in different populations?. *Gastroenterology*.

[11] Dixon WJ (1993). *BMDP Statistical Software Manual, 1992*.

[12] Feeley KM, Heneghan MA, Stevens FM, McCarthy CF (1998). Lymphocytic gastritis and coeliac disease: evidence of a positive association. *Journal of Clinical Pathology*.

[13] Luzza F, Mancuso M, Imeneo M (1999). *Helicobacter pylori* infection in children with celiac disease: prevalence and clinicopathologic features. *Journal of Pediatric Gastroenterology and Nutrition*.

[14] Hayat M, Arora DS, Dixon MF, Clark B, O'Mahony S (1999). Effects of *Helicobacter pylori* eradication on the natural history of lymphocytic gastritis. *Gut*.

[15] Niemelä S, Karttunen T, Kerola T, Karttunen R (1995). Ten year follow up study of lymphocytic gastritis: further evidence on *Helicobacter
pylori* as a cause of lymphocytic gastritis and corpus gastritis. *Journal of Clinical Pathology*.

[16] Oberhuber G, Bodingbauer M, Mosberger I, Stolte M, Vogelsang V (1998). High proportion of granzyme B-positive (activated) intraepithelial and lamina propria lymphocytes
in lymphocytic gastritis. *American Journal of Surgical Pathology*.

[17] De Giacomo C, Gianatti A, Negrini R (1994). Lymphocytic gastritis: a positive relationship with celiac disease. *Journal of Pediatrics*.

[18] Karttunen T, Niemelä S (1990). Lymphocytic gastritis and coeliac disease. *Journal of Clinical Pathology*.

[19] Lefrancois L, Puddington L, Ogra PL, Mestecky J, Lamm ME (1990). Basic aspects of intraepithelial lymphocytic immunobiology. *Mucosal Immunology*.

[20] Delves PJ, Roitt IM (2000). The immune system—second of two parts. *The New England Journal of Medicine*.

[21] Drut R, Drut RM (2004). Lymphocytic gastritis in pediatric celiac disease—immunohistochemical study
of the intraepithelial lymphocytic component. *Medical Science Monitor*.

[22] Bedoya A, Garay J, Sanzon F (2003). Histopathology of gastritis in *Helicobacter pylori*-infected children from populations at high and low gastric cancer risk. *Human Pathology*.

[23] Ernst PB, Takaishi H, Crowe SE (2001). *Helicobacter pylori* infection as a model for gastrointestinal immunity and chronic inflammatory diseases. *Digestive Diseases*.

[24] Santarelli L, Gabrielli M, Santoliquido A (2006). Interaction between *Helicobacter pylori* infection and untreated coeliac disease on gastric histological pattern. *Scandinavian Journal of Gastroenterology*.

[25] Anderson RP, van Heel DA, Tye-Din JA (2005). T cells in peripheral blood after gluten challenge in coeliac disease. *Gut*.

